# Combined analysis of preoperative circulating tumor cells status and tumor size to guide width of resection margin for solitary hepatocellular carcinoma

**DOI:** 10.3389/fonc.2026.1810914

**Published:** 2026-10-16

**Authors:** Jiansen Xie, Qijie Luo, Dongdong Huang, Feng Xia, Zhenheng Wu, Wei Zhou, Qiao Zhang, Feng Tian, Chi-Yi Liu, Zhihui Xu, Xian-Ting Jiao, Wei-Qiao Zhang, Mo Ali, Yi Zhou, Hengyi Gao

**Affiliations:** 1 Department of Hepatobiliary Surgery, Longhua District People's Hospital, Shenzhen, China; 2 Department of Hepatobiliary Surgery I, Zhongshan People's Hospital, Zhongshan, China; 3 Department of Liver Surgery, Tongji Hospital, Tongji Medical College, Huazhong University of Science and Technology, Wuhan, China; 4 Department of Hepatobiliary Surgery, Union Hospital, Fujian Medical University, Fuzhou, China; 5 Department of Hepatobiliary Surgery, Xiaogan Central Hospital, Xiaogan, China; 6 Graduate School, Guangdong Medical University, Zhanjiang, China; 7 Department of Anesthesiology I, Zhongshan People's Hospital, Zhongshan, China; 8 Department of Anesthesiology II, Zhongshan People's Hospital, Zhongshan, China; 9 Department of Anesthesiology, Shenzhen Longgang District People's Hospital, Shenzhen, China; 10 Intensive Care Unit, Zhongshan People's Hospital, Zhongshan, China; 11 Department of Hepatobiliary Surgery, The First Affiliated Hospital of Shaoyang University, Shaoyang, China

**Keywords:** circulating tumor cells, hepatocellular carcinoma, microvascular invasion, prognosis, resection margin, solitary HCC, tumor recurrence

## Abstract

**Background:**

The optimal resection margin width for hepatocellular carcinoma (HCC) remains controversial. The purpose of this study was to explore the value of preoperative circulating tumor cells (CTCs) status and tumor size in guiding the resection margin width of patients with HCC.

**Methods:**

This study was a multicenter retrospective study that included 383 patients who underwent resection of hepatocellular carcinoma (HCC). Preoperative circulating tumor cell (CTC) detection (CTC ≥ 1/CTC = 0) and imaging evaluation of tumor size (≤ 3 cm/> 3 cm) were performed in all patients. We aimed to compare recurrence-free survival (RFS) and overall survival (OS) between patients receiving narrow (< 1 cm) and wide (≥ 1 cm) resection margins.

**Results:**

Tumor diameter and CTC status were independent predictors of RFS and OS in the entire cohort. In patients with tumor diameter ≤ 3 cm, CTC-positive status correlated with poorer RFS but not OS. RFS and OS of narrow margin group and wide margin group were similar, and there was no statistical significance (P > 0.05). In the patients with tumor diameter > 3 cm and CTC-negative group, the RFS and OS of the narrow margin group were comparable to those of the wide margin group (P > 0.05). However, in patients with tumor diameter > 3 cm and CTC-positive groups, RFS and OS were better in the wide margin group than in the narrow margin group (P < 0.05).

**Conclusions:**

Using preoperative CTC status and tumor diameter as preoperative markers, combined analysis of CTC status and tumor diameter could help to determine surgical margin width in patients with solitary HCC. Narrow margins are indicated for patients with a tumor diameter ≤ 3 cm (regardless of CTC status) and for patients with a tumor diameter > 3 cm and CTC-negative. Whereas wide margins are preferred in patients with tumors > 3 cm in diameter and CTC-positive.

## Highlight

1. This study firstly found that the impact of CTC status on the prognosis of HCC patients is a stratified effect, especially the impact of CTC status on the prognosis of HCC patients with a diameter > 3 cm is more obvious.
2. This study is the first to combine preoperative CTC status and tumor diameter to guide surgical margin width in HCC patients. It is more accurate to screen out patients who need wide resection margins, so that those patients with low recurrence risk can avoid excessive liver parenchyma resection, which is in line with the concept of precision medicine.
3. This study is the first to suggest that for patients with a diameter > 3 cm and positive CTCs preoperatively, wide margins can reduce early postoperative recurrence compared with narrow margins.
4. Preoperative CTC detection and tumor diameter measurement are clinical information that can be obtained before surgery, and these two variables are objective and less invasive indicators, which can be widely promoted in clinical process.

## Introduction

Hepatocellular carcinoma (HCC) is the sixth most common malignancy worldwide and the third leading cause of cancer death (1, 2). With the improvement of people’s awareness of HCC surveillance and the improvement of the level of diagnosis, more and more early-stage HCC patients are detected in time (3). Currently, hepatectomy and liver transplantation(LT)are the main treatment options for HCC for early stage HCC (4–6). Due to the scarcity of organs, liver resection is ideal for HCC patients (5, 7). Even after surgical treatment, the 5-year recurrence-free survival (RFS) rate is only 50-70%, which severely reduces the long-term survival rate of HCC patients (6). Among them, early recurrence accounts for the vast majority, mainly because minimal residual disease (MRD) has not been effectively removed during surgery (8, 9). In the past, a large number of studies have shown that a wide enough resection margin can reduce early postoperative recurrence by reducing the occurrence of MRD (10–12), but an excessively wide resection margin may lead to liver failure after resection due to insufficient liver parenchyma (11, 13). At the same time, some scholars believe that wide resection margin does not improve the survival prognosis of HCC patients (14, 15). These different viewpoints could be attributed to varying probability of MRD in the residual liver after hepatectomy (14, 16). Therefore, it is recommended that the setting of surgical margins be determined according to whether the tumor is accompanied by MRD, especially the probability of microvascular invasion (MVI) (6, 14, 17).

A number of studies have reported that tumor size is not only associated with the incidence of MVI (18–20), but is also a stratification factor for identifying patients who may benefit from wide resection (12, 14, 21). However, a study by Poon et al. from Hong Kong showed that wide margins did not improve patient survival in different tumor diameter subgroups (15). These inconsistent studies suggest low reliability of using tumor size as the sole stratification factor to guide margin width (14). In our recent study, it has been confirmed that the status of CTC is highly correlated with the occurrence of MVI and could be an indicator to guide the choice of early HCC treatment (6, 22, 23). To make the study more objective, this study will explore whether tumor diameter combined with preoperative CTC status could be used to guide the width of surgical margins.

## Materials and methods

### Patient with HCC

This study recruited HCC patients who underwent preoperative CTC testing at the Department of Hepatobiliary Surgery of Zhongshan People’s Hospital and Shenzhen Long-Hua People’s Hospital, with the following inclusion criteria: (1) patients diagnosed with HCC by postoperative pathology, (2) not receiving any form of treatment intervention before operation, (3) radical tumor resection (R0) that is, negative macroscopic and microscopic tumor resection margins, (4) complete serological and imaging data, (5) tumor diameter not more than 5 cm, and (6) single tumor. Exclusion criteria were (1) patients younger than 18 years of age, (2) presence of vascular tumor thrombus and distant metastasis, (3) patients with Child-Pugh grade C liver function, (4) no severe vital organ dysfunction, (5) patients who underwent palliative resection, and (6) loss of postoperative follow-up data. The Ethics Committee of Zhongshan People’s Hospital and Shenzhen Long-Hua People’s Hospital have approved the retrospective study, all patients have signed an informed consent form.

### Data collection

All patients underwent abdominal enhanced CT or MRI, chest CT or X-ray scan. Laboratory tests include blood routine, liver and kidney function, coagulation function, hepatitis B surface antigen, hepatitis C antibody, AFP, and other examinations. The basic data of the included study population, such as gender, age, hepatitis B surface antigen, hepatitis C antibody, AFP level, alanine aminotransferase (ALT), aspartate aminotransferase (AST), γ-glutamyl aminotransferase (GGT), alkaline phosphatase (ALP), creatinine (Cr), albumin (Alb), total bilirubin (TIBL), international normalized ratio (INR), presence of cirrhosis, Child-Pugh grade, presence of MVI, maximum tumor diameter. The continuous variables are transformed into binary variables, and the cut-off value is the upper and lower lines of the recognized normal value (2, 22, 24). All above clinicopathological indicators were collected for subsequent statistical analyses.

### CTC isolation and detection

Three days before surgery, we drew 5 mL of peripheral blood as a sample for inspection and strictly processed the sample according to the manufacturer’s instructions. The detection of CTCs was performed by the “Cyttel” method (Jiangsu, China), whose principles include the negative immunomagnetic particle method and immunofluorescence *in situ* hybridization (im-FISH) (25–27). The former mainly uses immunomagnetic particles (anti-CD45 antibody-conjugated magnetic beads) as the carrier, through the principle of antigen–antibody reaction, combined with centrifugation technology, to remove leukocytes from the blood *in vitro* to separate rare cells. Then, the samples were fixed on glass slides, dehydrated with ethanol, dried, and then hybridized with chromosome centromere probe No. 1 and chromosome centromere probe No. 8. Finally, 4-diamidine-2-phenylindole (DAPI) staining was added to seal the samples, and the CTCs were observed and counted under a fluorescence microscope (Microprofit, China) (28). CTC count ≥1/5 mL was defined as CTC-positive (22, 25, 29, 30).

### Surgical resection

Each patient was evaluated preoperatively, and a corresponding surgical plan was formulated according to the patient’s tumor location, tumor size, and the relationship with the adjacent large blood vessels in the liver. Surgical resection was divided into laparoscopic hepatectomy and open hepatectomy. The scope of surgical resection included: segmentectomy, and hemihepatectomy. The basic procedure of the operation is as follows: First, perform anesthesia and routine disinfection. After entering the abdominal cavity, first observe whether the tumor has distant metastasis to the adjacent organs, and use the corresponding instruments to separate the corresponding tissue structure. This is followed by intermittent occlusion of the first porta hepatis and selective blockade of the second porta hepatis if necessary. The corresponding hepatic segment was divided according to the tumor location. After finding no residual tumor at the resection margin, blood flow stoppage was performed at the resection margins, the first and second hepatic hilum were released, and finally the abdomen was closed. All patients enrolled in this study underwent radical R0 resection, and cases with R1/R2 resection were excluded according to our inclusion and exclusion criteria. All patients enrolled in this study underwent radical R0 resection, and cases with R1/R2 resection were excluded according to our inclusion and exclusion criteria.Surgical resection procedures are performed by senior surgeons who are senior associate chief physicians or above. The pathologist-reported margin width was defined as the shortest macroscopic distance from the edge of the tumor to the line of transection (6). Wide/narrow margins were defined as the cut-off value of 1 cm, the incision margin width ≥ 1 cm was defined as wide margin, and < 1 cm was defined as narrow margin (5, 10, 11, 15).

### Follow-up

Each follow-up visit for all patients included AFP, routine blood tests, liver and kidney function tests, and coagulation function tests. Enhanced CT or MRI of the abdomen, chest CT, and bone scan were arranged if tumor residue and signs of tumor recurrence were suspected. The first postoperative follow-up visit was performed one month after the operation. The follow-up frequency was once every 2–3 months within six months after the operation, once every 3–4 months within 6–24 months after the operation, and once every 4–6 months after 24 months after the operation. After a recurrence of HCC, treatment options were chosen according to the recurrence and the patient’s general condition. Treatment options included surgical re-resection, radiofrequency ablation (RFA), percutaneous ethanol injection (PEI), TACE, targeted drugs, immunotherapy, and even liver transplantation. OS was defined as the date of surgery until patient’s death or last follow-up, and RFS was defined as the date of surgery until patients’ signs of recurrence or last follow-up. Recurrence was classified as early recurrence and late recurrence using a cut-off value of 24 months (2, 22, 31, 32). The last day of follow-up was August 1, 2022.

### Statistical analysis

Continuous variables with normal distribution were presented as mean ± standard deviation (SD), while non-normally distributed continuous data were reported as median (interquartile range, IQR), and categorical variables were expressed as numbers (n) or percentages (%) of patients. The t-test or Mann-Whitney U test was used to compare two groups of continuous variables, and the χ² or Fisher’s exact test was used to compare two groups of categorical variables. The survival curves of OS and RFS of patients were drawn by the Kaplan-Meier method, and the OS and RFS of the wide-margin group and narrow-margin group were compared using the Log-rank test. We also used Landmark analysis to assess outcomes for early recurrence (≤24 months postoperatively) and late recurrence (>24 months). Univariate and multivariate Cox regression models were used to analyze the independent risk factors of related clinical variables for RFS and OS in patients. All statistics and graphics for this study were done in R software (version 3.62). A P value <0.05 was considered to be statistically significant.

## Results

### Patient characteristics

In this study, a total of 383 HCC patients were included in this study, of which 148 patients were CTC-positive and 235 were CTC-negative. The surgical methods included 140 patients (36.6%) in the wide margin group and 243 patients (63.4%) in the narrow margin group. The median follow-up time was 30 months (IQR [18, 45]). During the follow-up period, tumor recurrence occurred in 183 patients (47.8%) and death occurred in 92 patients (24.0%). Other clinical variables are presented in **Table 1**.

**Table 1:** Baseline characteristics of HCC patients for the entire cohort.

Variable		n = 383	
		n	%
Age (years)	<60	140	36.6
	≥60	243	63.4
Sex	Female	111	29.0
	Male	272	71.0
HBV	No	40	10.4
	Yes	343	89.6
HCV	No	367	95.8
	Yes	16	4.2
Cirrhosis	No	120	31.3
	Yes	263	68.7
Child-Pugh	A	346	90.3
	B	37	9.7
ALT (U/L)	<50	309	80.7
	≥50	74	19.3
AST (U/L)	<40	270	70.5
	≥40	113	29.5
GGT (U/L)	<60	265	69.2
	≥60	118	30.8
ALP (U/L)	<125	326	85.1
	≥125	57	14.9
Alb (g/L)	<35	71	18.5
	≥35	312	81.5
TBIL (µmol/L)	<20.4	307	80.2
	≥20.4	76	19.8
CR (µmol/L)	<104	377	98.4
	≥104	6	1.6
INR	<1.20	357	93.2
	≥1.20	26	6.8
AFP (µg/mL)	<400	263	68.7
	≥400	127	31.2
Grade	I+II	318	83.0
	III+IV	65	17.0
MVI	No	269	70.2
	Yes	114	29.8
CTC status	Negative	235	61.4
	Positive	148	38.6
Tumor size	≤ 30mm	179	46.7
	> 30 mm	204	53.3
Surgical methods	Narrow	243	63.4
	Wide	140	36.6

AST, aspartate aminotransferase; ALT, alanine aminotransferase; GGT, gamma glutamyl transpeptidase; ALP, alkaline phosphatase; Alb, albumin; TIBL, total bilirubin; DIBL, direct bilirubin; CR, creatinine; INR, international normalized ratio; AFP, alpha fetoprotein; HBV, hepatitis B virus; HCV, hepatitis C virus; CTC, circulating tumor cells.

### CTC status and tumor size were independent prognostic predictors for patients with solitary HCC

Using survival curves drawn by the Kaplan Meier method, we found that the OS (P < 0.05; **Supplementary Figure 1A**) and RFS (P < 0.05; **Supplementary Figure 1B**) of the CTC-positive group were worse than those of the CTC-negative group. Through univariate and multivariate Cox regression analysis, we found that tumor diameter > 3 cm and CTC status were independent risk factors for OS and RFS(P < 0.05; **Supplementary Tables 1**, **2**).

### Subgroup analysis based on tumor size to assess the impact of CTC status on survival prognosis

Using the tumor diameter of 3 cm as the cut-off value, the effect of CTC status on survival and prognosis of patients in different tumor size subgroups was explored. The results showed that CTC status was a risk factor for survival prognosis of the population with HCC whose tumor diameter was greater than 3 cm (P < 0.05; **Supplementary Figures 1E, F**). However, in the HCC population with tumor diameter ≤3 cm, we only found that the RFS in the CTC-positive group was worse than that in the CTC-negative group (P < 0.05; **Supplementary Figure 1D**), while the OS was roughly equivalent (P > 0.05; **Supplementary Figure 1C**).

In addition to this, we also analyzed the relationship between CTC status and postoperative recurrence patterns using the landmark method. With a cutoff of 24 months, the results showed that there was a stratified effect of CTC status on the survival prognosis of patients. CTC-positive was associated with early postoperative recurrence (P < 0.05; **Supplementary Figures 2A, C**) but not with late recurrence (P > 0.05; **Supplementary Figures 2A, C**) in both the overall and tumor size > 3 cm groups. But in the tumor size ≤3 cm group, CTC status was not associated with recurrence pattern (P > 0.05; **Supplementary Figure 2B**).

### Combining tumor size and CTC status as stratification factors to assess the effect of margin width on postoperative prognosis and recurrence

#### For HCC patients with tumor diameter ≤3 cm, regardless of CTC status, margin width had no effect on RFS and OS

For HCC patients with tumor size ≤3 cm, a total of 179 patients were included, including 111 (62.1%) in the narrow margin group and 68 (37.9%) in the wide margin group. We compared the general clinicopathological data between the wide margin group and the narrow margin group, and found that the general clinicopathological data were not statistically significant (**Table 2**; P>0.05). At the same time, we compared the effect of different margin widths on RFS and OS of patients. The study showed that regardless of the CTC status, the margin width was not associated with the survival prognosis of patients (**Figures 1A–F**; P>0.05).

**Table 2:** Comparison of clinicopathological characteristics of HCC patients with tumor diameter ≤ 3 cm in different resection margin groups.

Variable	n=179	Narrow (n=111)	Wide(n=68)	*P*
Age (years)	<60	35 (31.5)	29 (42.6)	0.179
	≥60	76 (68.5)	39 (57.4)	
Sex	Female	25 (22.5)	22 (32.4)	0.202
	Male	86 (77.5)	46 (67.6)	
HBV	No	9 (8.1)	10 (14.7)	0.254
	Yes	102 (91.9)	58 (85.3)	
HCV	No	108 (97.3)	64 (94.1)	0.504
	Yes	3 (2.7)	4 (5.9)	
Cirrhosis	No	29 (26.1)	25 (36.8)	0.181
	Yes	82 (73.9)	43 (63.2)	
Child-Pugh	A	101 (91.0)	61 (89.7)	0.982
	B	10 (9.0)	7 (10.3)	
ALT (U/L)	<50	85 (76.6)	53 (77.9)	0.978
	≥50	26 (23.4)	15 (22.1)	
AST (U/L)	<40	80 (72.1)	45 (66.2)	0.505
	≥40	31 (27.9)	23 (33.8)	
GGT (U/L)	<60	80 (72.1)	47 (69.1)	0.800
	≥60	31 (27.9)	21 (30.9)	
ALP (U/L)	<125	88 (79.3)	57 (83.8)	0.578
	≥125	23 (20.7)	11 (16.2)	
Alb (g/L)	<35	19 (17.1)	13 (19.1)	0.890
	≥35	92 (82.9)	55 (80.9)	
TBIL (µmol/L)	<20.4	86 (77.5)	59 (86.8)	0.180
	≥20.4	25 (22.5)	9 (13.2)	
CR (µmol/L)	<104	110 (99.1)	67 (98.5)	1.000
	≥104	1 (0.9)	1 (1.5)	
INR	<1.20	104 (93.7)	62 (91.2)	0.739
	≥1.20	7 (6.3)	6 (8.8)	
AFP (µg/mL)	<400	80 (72.1)	48 (70.6)	0.966
	≥400	31 (27.9)	20 (29.4)	
Grade	I+II	98 (88.3)	59 (86.8)	0.947
	III+IV	13 (11.7)	9 (13.2)	
MVI	No	94 (84.7)	59 (86.8)	0.869
	Yes	17 (15.3)	9 (13.2)	
Diameter (mean (SD))		22.83 (5.16)	21.97 (5.57)	0.296
CTC (%)	N	78 (70.3)	47 (69.1)	1.000
	P	33 (29.7)	21 (30.9)	

AST, aspartate aminotransferase; ALT, alanine aminotransferase; GGT, gamma glutamyl transpeptidase; ALP, alkaline phosphatase; Alb, albumin; TBIL, total bilirubin; DIBL, direct bilirubin; CR, creatinine; INR, international normalized ratio; AFP, alpha fetoprotein; HBV, hepatitis B virus; HCV, hepatitis C virus; CTC, circulating tumor cells.

**Figure 1:**
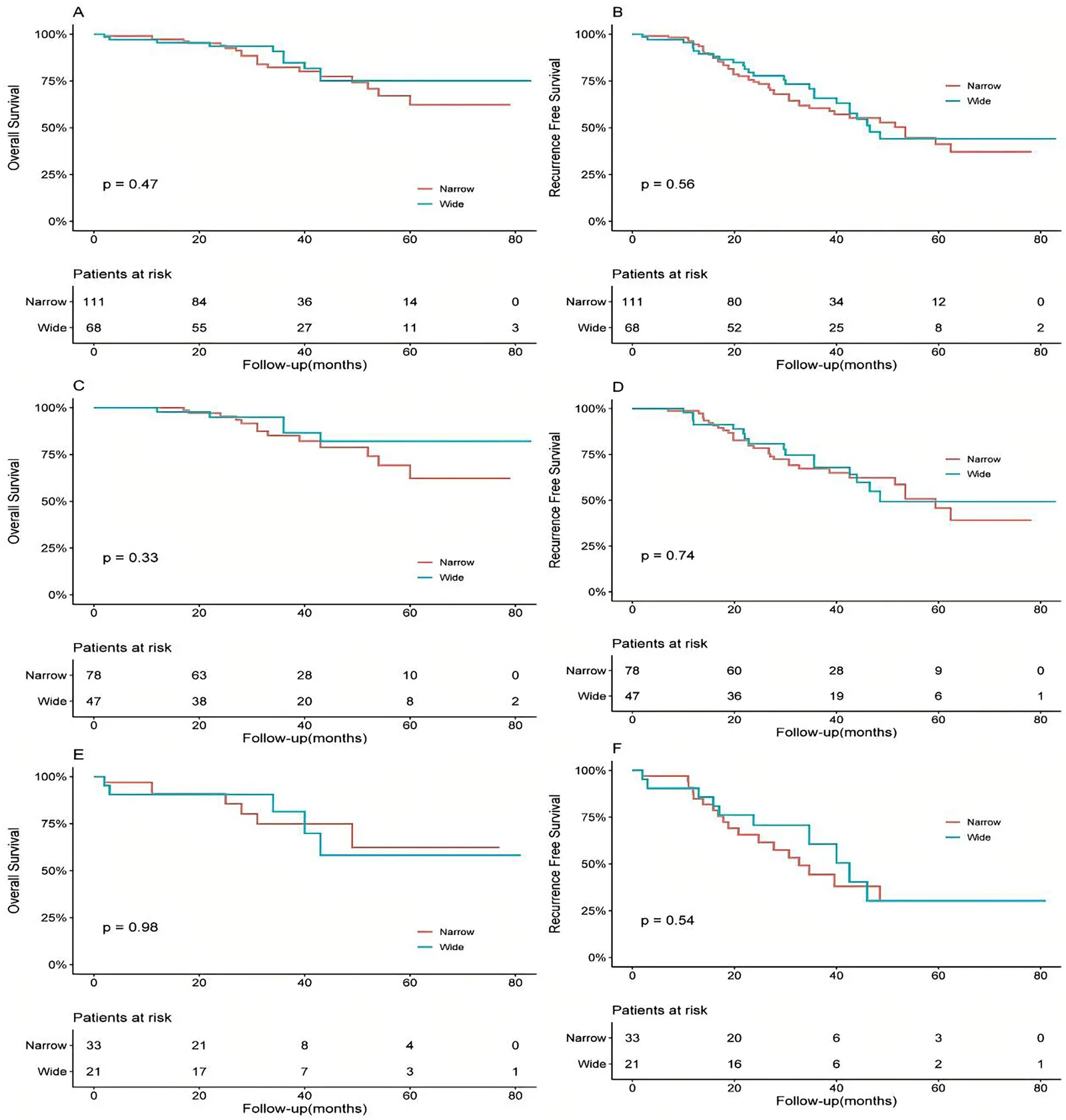
Survival outcomes of the HCC patients with tumor size≤ 3 cm and CTC status-based subgroups divided by margin width. Recurrence-free survival and overall survival curves for HCC patients with tumor size ≤ 3 cm **(A, B)**, patients with CTC-negative **(C, D)**, and those with CTC-positive **(E, F)** divided by margin width.

Univariate and Multivariate Cox regression analysis found that the RFS and OS of HCC with tumor size ≤3 cm were not related to the width of the resection margin (**Supplementary Tables 3**, **4**; P>0.05).

#### For HCC patients with tumor diameter > 3 cm, there was a stratified effect of margin width on RFS and OS

For HCC patients with tumor size > 3 cm, a total of 204 patients were included, including 132 (64.7%) in the narrow margin group and 72 (35.3%) in the wide margin group. We compared the general clinicopathological data of the wide margin group and the narrow margin group. There was no statistically significant difference in general clinicopathological variables between the two groups, and they were comparable (**Table 3**, P>0.05). We also compared the effect of different margin widths on RFS and OS in patients. The results showed that there was a stratified effect on the effect of margin width on the survival prognosis of patients (**Figures 2A–F**). Wide margins and narrow margins were only beneficial in the CTC-positive group (**Figures 2A, B, E, F**; P<0.05), but not in CTC-negative patients (**Figures 2C, D**; P>0.05).

**Table 3:** Comparison of clinicopathological characteristics of HCC patients with tumor diameter > 3 cm in different resection margin groups.

Variable		Narrow (n=132)	Wide(n=72)	*P*
Age (years)	<60	48 (36.4)	28 (38.9)	0.838
	≥60	84 (63.6)	44 (61.1)	
Sex	Female	44 (33.3)	20 (27.8)	0.510
	Male	88 (66.7)	52 (72.2)	
HBV	No	17 (12.9)	4 (5.6)	0.160
	Yes	115 (87.1)	68 (94.4)	
HCV	No	126 (95.5)	69 (95.8)	1.000
	Yes	6 (4.5)	3 (4.2)	
Cirrhosis	No	43 (32.6)	23 (31.9)	1.000
	Yes	89 (67.4)	49 (68.1)	
Child-Pugh	A	117 (88.6)	67 (93.1)	0.442
	B	15 (11.4)	5 (6.9)	
ALT (U/L)	<50	116 (87.9)	55 (76.4)	0.054
	≥50	16 (12.1)	17 (23.6)	
AST (U/L)	<40	96 (72.7)	49 (68.1)	0.588
	≥40	36 (27.3)	23 (31.9)	
GGT (U/L)	<60	91 (68.9)	47 (65.3)	0.706
	≥60	41 (31.1)	25 (34.7)	
ALP (U/L)	<125	114 (86.4)	67 (93.1)	0.225
	≥125	18 (13.6)	5 (6.9)	
Alb (g/L)	<35	27 (20.5)	12 (16.7)	0.637
	≥35	105 (79.5)	60 (83.3)	
TBIL (µmol/L)	<20.4	110 (83.3)	52 (72.2)	0.090
	≥20.4	22 (16.7)	20 (27.8)	
CR (µmol/L)	<104	128 (97.0)	72 (100.0)	0.335
	≥104	4 (3.0)	0 (0.0)	
INR	<1.20	122 (92.4)	69 (95.8)	0.514
	≥1.20	10 (7.6)	3 (4.2)	
AFP (µg/mL)	<400	81 (61.4)	54 (75.0)	0.07
	≥400	51 (38.6)	18 (25.0)	
Grade	I+II	102 (77.3)	59 (81.9)	0.547
	III+IV	30 (22.7)	13 (18.1)	
MVI	No	69 (52.3)	47 (65.3)	0.100
	Yes	63 (47.7)	25 (34.7)	
Diameter (mean (SD))		39.90 (5.19)	39.54 (5.85)	0.651
CTC (%)	N	66 (50.0)	44 (61.1)	0.169
	P	66 (50.0)	28 (38.9)	

AST, aspartate aminotransferase; ALT, alanine aminotransferase; GGT, gamma glutamyl transpeptidase; ALP, alkaline phosphatase; Alb, albumin; TBIL, total bilirubin; DIBL, direct bilirubin; CR, creatinine; INR, international normalized ratio; AFP, alpha fetoprotein; HBV, hepatitis B virus; HCV, hepatitis C virus; CTC, circulating tumor cells.

**Figure 2:**
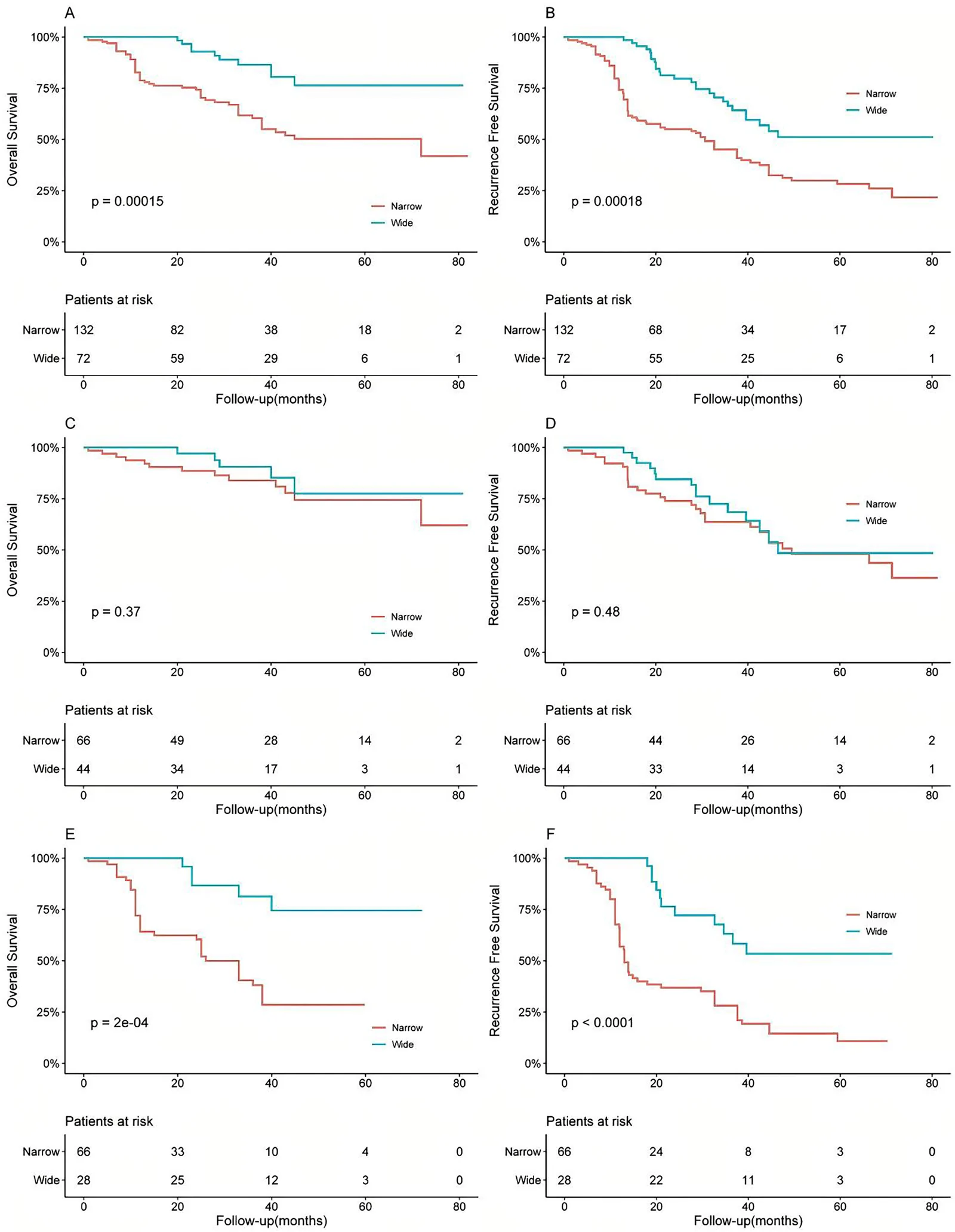
Survival outcomes of the HCC patients with tumor size> 3 cm and CTC status-based subgroups divided by margin width. Recurrence-free survival and overall survival curves for HCC patients with tumor size> 3 cm **(A, B)**, patients with CTC-negative **(C, D)**, and those with CTC-positive **(E, F)** divided by margin width.

Univariate and multivariate Cox regression analysis also found that in HCC patients with tumor size > 3 cm and CTC-negative group, margin width was not an independent risk factor for RFS and OS (**Supplementary Table 5**; P>0.05). For HCC patients with tumor size > 3 cm and CTC-positive group, margin width was an independent risk factor for RFS and OS. (**Supplementary Table 6**; P<0.05).

#### For HCC patients with tumor diameters > 3 cm, there was a stratified effect of margin width on postoperative tumor recurrence patterns

We used the Landmark method to analyze the effect of margin width on postoperative recurrence of HCC with tumor size > 3 cm. The results showed that compared with narrow margins, wide margins could reduce early postoperative recurrence (P<0.05; **Figures 3A, C**) but not late recurrence (P>0.05; **Figures 3A, C**) in the CTC-positive group and overall patients. In the CTC-negative group, the width of the resection margin was not associated with the postoperative recurrence pattern (P>0.05; **Figure 3B**).

**Figure 3:**
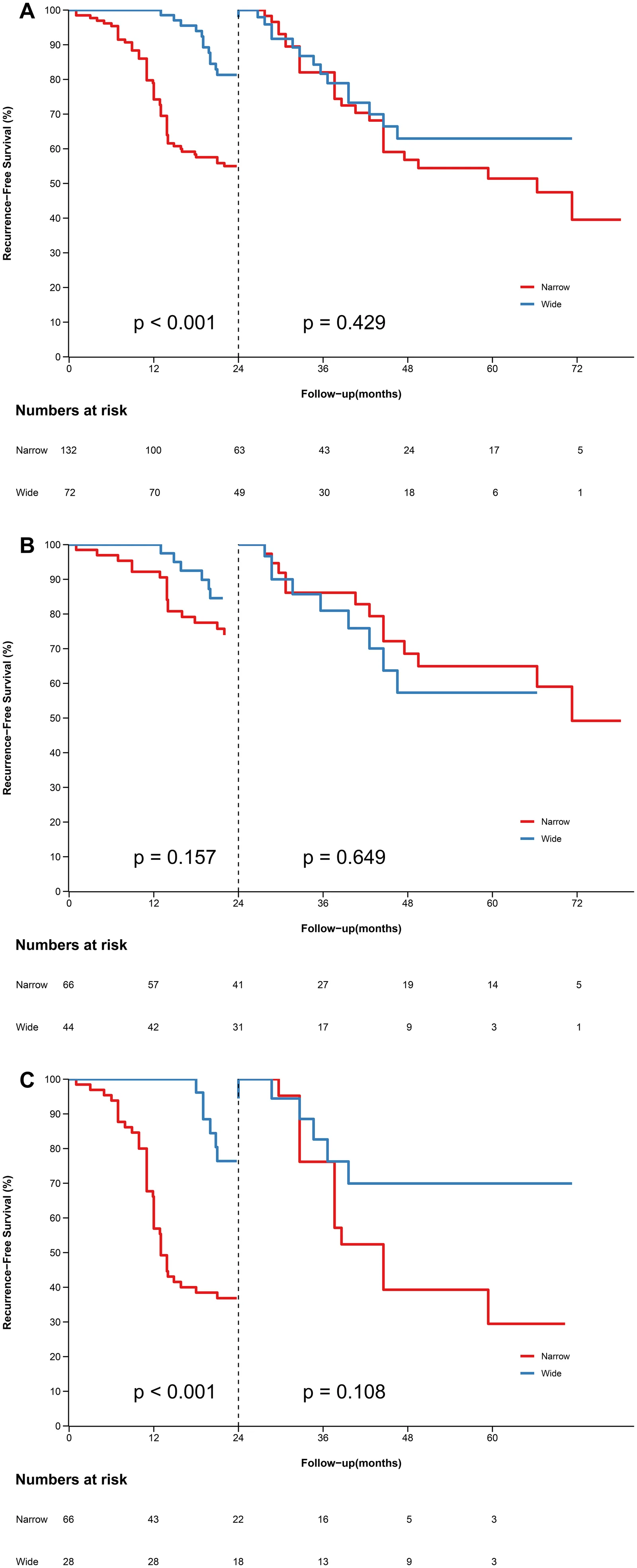
Analysis of the effect of margin width on early and late postoperative recurrence for HCC patients with tumor size> 3 cm **(A)**, patients with CTC-negative **(B)**, and those with CTC-positive **(C)** by landmark method.

### CTC status correlates with microvascular infiltration

For the population undergoing surgery, the positive rates of CTCs in tumor sizes ≤3 cm and > 3 cm were 14.5% and 43.1%, respectively. No matter in the tumor size ≤ 3 cm or > 3 cm group, CTC-positive was correlated with microvascular invasion (MVI) (P<0.05; **Table 4**). No significant correlations were observed between CTC status and other clinicopathological variables, including AFP level and tumor differentiation grade (P>0.05; **Table 4**).

**Table 4:** Relationship between CTC status with clinical characteristics for patients in tumor sizes ≤3 cm and > 3 cm.

Variable		Tumor Size ≤ 3 cm		*P*	Tumor Size> 3 cm		*P*
		CTC=0 (n=125)	CTC≥1(n=54)		CTC=0 (n=110)	CTC≥1(n=94)	
Age (years)	<60	40 (32.0)	24 (44.4)	0.154	37 (33.6)	39 (41.5)	0.312
	≥60	85 (68.0)	30 (55.6)		73 (66.4)	55 (58.5)	
Sex	Female	30 (24.0)	17 (31.5)	0.390	31 (28.2)	33 (35.1)	0.362
	Male	95 (76.0)	37 (68.5)		79 (71.8)	61 (64.9)	
HBV	No	11 (8.8)	8 (14.8)	0.350	7 (6.4)	14 (14.9)	0.077
	Yes	114 (91.2)	46 (85.2)		103 (93.6)	80 (85.1)	
HCV	No	121 (96.8)	51 (94.4)	0.744	107 (97.3)	88 (93.6)	0.355
	Yes	4 (3.2)	3 (5.6)		3 (2.7)	6 (6.4)	
Cirrhosis	No	33 (26.4)	21 (38.9)	0.135	38 (34.5)	28 (29.8)	0.566
	Yes	92 (73.6)	33 (61.1)		72 (65.5)	66 (70.2)	
Child-Pugh	A	114 (91.2)	48 (88.9)	0.837	102 (92.7)	82 (87.2)	0.281
	B	11 (8.8)	6 (11.1)		8 (7.3)	12 (12.8)	
ALT (U/L)	<50	95 (76.0)	43 (79.6)	0.736	90 (81.8)	81 (86.2)	0.515
	≥50	30 (24.0)	11 (20.4)		20 (18.2)	13 (13.8)	
AST (U/L)	<40	85 (68.0)	40 (74.1)	0.525	72 (65.5)	73 (77.7)	0.078
	≥40	40 (32.0)	14 (25.9)		38 (34.5)	21 (22.3)	
GGT (U/L)	<60	86 (68.8)	41 (75.9)	0.433	72 (65.5)	66 (70.2)	0.566
	≥60	39 (31.2)	13 (24.1)		38 (34.5)	28 (29.8)	
ALP (U/L)	<125	102 (81.6)	43 (79.6)	0.920	100 (90.9)	81 (86.2)	0.398
	≥125	23 (18.4)	11 (20.4)		10 (9.1)	13 (13.8)	
Alb (g/L)	<35	19 (15.2)	13 (24.1)	0.226	16 (14.5)	23 (24.5)	0.106
	≥35	106 (84.8)	41 (75.9)		94 (85.5)	71 (75.5)	
TBIL (µmol/L)	<20.4	100 (80.0)	45 (83.3)	0.753	85 (77.3)	77 (81.9)	0.520
	≥20.4	25 (20.0)	9 (16.7)		25 (22.7)	17 (18.1)	
CR (µmol/L)	<104	123 (98.4)	54 (100.0)	0.873	109 (99.1)	91 (96.8)	0.506
	≥104	2 (1.6)	0 (0.0)		1 (0.9)	3 (3.2)	
INR	<1.20	117 (93.6)	49 (90.7)	0.717	105 (95.5)	86 (91.5)	0.385
	≥1.20	8 (6.4)	5 (9.3)		5 (4.5)	8 (8.5)	
AFP (µg/mL)	<400	95 (76.0)	33 (61.1)	0.065	79 (71.8)	56 (59.6)	0.090
	≥400	30 (24.0)	21 (38.9)		31 (28.2)	38 (40.4)	
Grade	I+II	113 (90.4)	44 (81.5)	0.156	92 (83.6)	69 (73.4)	0.107
	III+IV	12 (9.6)	10 (18.5)		18 (16.4)	25 (26.6)	
MVI	No	112 (89.6)	41 (75.9)	**0.031**	76 (69.1)	40 (42.6)	**<0.001**
	Yes	13 (10.4)	13 (24.1)		34 (30.9)	54 (57.4)	
Diameter (mean (SD))		22.56 (5.29)	22.37 (5.44)	0.827	40.43 (5.78)	39.01 (4.88)	0.062
Group (%)	Narrow	78 (62.4)	33 (61.1)	1.000	66 (60.0)	66 (70.2)	0.169
Wide	47 (37.6)	21 (38.9)	44 (40.0)		28 (29.8)		

AST, aspartate aminotransferase; ALT, alanine aminotransferase; GGT, gamma glutamyl transpeptidase; ALP, alkaline phosphatase; Alb, albumin; TBIL, total bilirubin; DIBL, direct bilirubin; CR, creatinine; INR, international normalized ratio; AFP, alpha fetoprotein; HBV, hepatitis B virus; HCV, hepatitis C virus; MVI, Microvascular Infiltration.

Bold values indicate statistically significant differences (P < 0.05).

## Discussion

Surgical resection remains the mainstay of treatment for patients diagnosed with HCC (33), and although R0 resection may improve long-term outcomes (34), its postoperative recurrence rate remains as high as 70% (35). The main reason for this may be that the R0 resection also includes a large number of cases with narrow margins. From a pathological point of view, the lesions of hepatocellular carcinoma are not only composed of the main lesion, but also include the surrounding MVI (36). The narrow resection margin cannot completely remove the MVI around the main lesion (6). Therefore, adequate margin width is crucial to reduce postoperative recurrence (11, 14, 37, 38). On the other hand, preservation of non-neoplastic liver parenchyma is also an important consideration, especially in cirrhotic hepatectomy. The advantages of sparing as much liver parenchyma as possible include not only reducing the incidence of postoperative liver failure (15, 39, 40), but also increasing the chance of multimodal therapy and repeat resection in the event of tumor recurrence (11, 15, 16). Therefore, there is an urgent need to screen out those HCC populations that do not require wide margins, so as to preserve more liver parenchyma. Essentially, the advantage of wide margins over narrow margins is that Minimal Residual Disease (MRD) could be effectively treated. If MRD can be effectively predicted preoperatively, it will guide us to choose a wide margin (41). Previous studies have shown that wide margins can reduce early recurrence in patients with MVI compared with narrow margins, but this advantage does not exist in patients without MVI (10, 41). This demonstrates the value of MRD in guiding the choice of margin width. Although a large number of past studies have used clinical tumor characteristics and imaging to predict the incidence of MVI (42–48), their imaging protocols lack standardized criteria, and predictive models show limited accuracy, which restricts broad clinical implementation (49).

The researchers from the Eastern Hepatobiliary Surgery Hospital successfully used the two variables of tumor capsule and tumor diameter to guide the surgical margin width of HCC (14). It is undeniable that the determination of the integrity of the tumor capsule is not only subjective, but also affected by many factors such as equipment (17). This greatly limits its application in clinical practice. Therefore, it is necessary to seek simpler and easier markers to guide margin width.

CTCs are malignant tumor cells that invade into the peripheral blood via the form of Epithelial-Mesenchymal Transition (EMT), which can serve as a prognostic indicator of disease progression and it is associated with the aggressiveness of tumors (50–52).

In recent years, with the in-depth understanding of CTCs in the field of HCC, the close relationship between CTC status and MVI has been confirmed (2, 6, 22–24). This study shows that there is a positive correlation between CTC status and the incidence of MVI. That is CTC-positive patients have a higher probability of MVI than CTC-negative groups, which is also consistent with the conclusions of previous studies (2, 6, 22–24). Similar to past studies (2, 53, 54), in the overall population, we found that CTC status and tumor diameter were independent risk factors for survival prognosis in patients with solitary HCC. However, CTC status has a stratified effect on prognosis of patients. That is, CTC status is an independent risk factor for prognosis in HCC patients with tumor diameter > 3 cm, but it has no significant effect on the OS of HCC patients with tumor diameter ≤ 3 cm. A large-scale retrospective study from a multicenter showed that, the distance of MVI from the main lesion >1 cm (M2) accounted for 28% of the overall MVI for patients with tumor diameter ≤3 cm (55). In our study, the rate of MVI (including M1 and M2) was only 14.5% for patients with tumor diameter ≤3 cm, which means that the incidence of M2 may only account for less than 4%. Theoretically, a narrow margin can remove MVI regardless of CTC status, for patients with tumor diameter ≤3 cm. Our results showed that no difference was found in OS and RFS between wide and narrow margins, regardless of CTC-positive or CTC-negative, which confirmed the above conjecture. Similar conclusions have also been reported in the past (14, 38). Sasaki, K. et al. believed that the MVI rate of HCC with tumor size ≤ 3 cm was low, and its distribution range was quite limited, and the width of the resection margin had little effect on the prognosis of patients (38). A retrospective study by Zhou et al. concluded that for HCC with tumor size ≤3 cm, expanding the width of the resection margin did not improve the RFS and OS of the patients (14). Accordingly, surgeons do not need to pursue extended wide resection margins for solitary HCC ≤ 3 cm.

On the other hand, this study showed that the MVI rate for HCC with a diameter of > 3 cm was 43%, which was significantly higher than that of HCC ≤ 3 cm, and was similar to that of Pawlik, T. M. et al (20). Our study also found that wide margins had significantly better RFS and OS than narrow margins, especially in the CTC-positive group. In addition, we observed that in the CTC-positive group, wide resection margins could reduce the incidence of early postoperative recurrence. The above conclusions also corroborate the study of Zhou et al (23). Why does this stratification occur? We believe that it can be explained by two factors, the incidence and severity of MVI. First, the incidence of MVI is significantly higher in HCC patients with tumor size > 3 cm than tumor diameter ≤ 3 cm (20, 55). Second, in terms of the severity of MVI, both tumor diameter and CTC status were positively correlated with the distribution of MVI (6, 55). Therefore, for patients with tumor diameter > 3 cm and CTC-positive, narrow margins cannot effectively remove MVI, resulting in residual tumor and more prone to early tumor recurrence. In summary, wide resection margins could be a favorable option for patients with tumor diameter > 3 cm and positive CTCs, based on our retrospective findings. Or when the tumor is too large, preoperative adjuvant should be used to reduce the tumor burden and pursue a wide resection margin to improve the survival prognosis of patients. For patients with tumor diameter ≤ 3 cm or > 3 cm and CTC-negative, narrower margins can be safely performed without affecting prognosis. The indicators used in this study are all available through imaging and peripheral blood before operation, and the indicators are objective, less traumatic and strong operability, which are easy to be popularized and applied in clinic and help guide surgeons to make surgical decisions.

Although this study provides a new strategy for the guidance of margin width in hepatectomy, it does have certain limitations. 1. This study is a retrospective study, there are many potential biases and confounding factors that cannot be eliminated, and the conclusions of the study need to be confirmed by a prospective study with a larger sample size. 2. Our research population is mainly the Chinese population. The vast majority of patients have hepatitis B virus-related hepatocellular carcinoma. Whether the conclusion is suitable for non-Asian populations, as well as patients with hepatitis C and alcohol-related HCC, is still unknown. 3. There may be temporal heterogeneity in the detection of CTCs, and the number of CTCs in the peripheral blood of tumors in different periods may be different (56). The conclusion of this study may only be suitable for HCC patients whose tumor diameters are less than or equal to 5 cm. 4. The cost of CTCs detection is high, and it cannot be performed as a routine test in some areas with poor economic conditions, but we believe that with the improvement of detection methods, the cost of detection can be reduced to be more commonly used in clinical practice. 5. The median follow-up time of this cohort was only 30 months, which was insufficient to draw robust long-term overall survival conclusions beyond 5 years. Further long-term follow-up prospective studies are required to validate the long-term survival discrepancies of different resection margin strategies.

In conclusion, CTC status and tumor diameter based on preoperative indicators were independent prognostic predictors in patients with solitary HCC with tumor diameter ≤5 cm who underwent hepatectomy. Combined analysis of CTC status and tumor diameter provides a convenient decision-making tool to guide surgical margin width. HCC patients with tumor diameter ≤ 3 cm (regardless of preoperative CTC status) and HCC patients with tumor diameter > 3 cm and CTC-negative are candidates for narrow margin resection without affecting prognosis. In contrast, wide margins are may be preferentially consideredfor patients with tumors > 3 cm in diameter and CTC-positive. Therefore, we suggest that surgeons should pay attention to preoperative CTC status and tumor diameter to develop safe and reasonable surgical strategies for HCC patients.

## Data Availability

The raw data supporting the conclusions of this article will be made available by the authors, without undue reservation.

## Supplementary material

Supplementary Figure 1 Survival outcomes of the entire cohort and tumor size-based subgroups divided by CTC status. Recurrence-free survival and overall survival curves for the entire cohort **(A, B)**, patients with tumor size ≤ 3 cm **(C, D)**, and those with tumor size > 3 cm **(E, F)** divided by CTC status.

Supplementary Figure 2 Analysis of the effect of CTC status on early and late postoperative recurrence entire cohort **(A)**, patients with tumor size ≤ 3 cm **(B)**, and those with tumor size > 3 cm **(C)** by landmark method.

Supplementary Table 1 Univariate and multivariate Cox regression analysis was used to identify independent risk factors for RFS in overall patients. AST, aspartate aminotransferase; ALT, alanine aminotransferase; GGT, gamma glutamyl transpeptidase; ALP, alkaline phosphatase; Alb, albumin; TIBL, total bilirubin; DIBL, direct bilirubin; CR, creatinine; INR, international normalized ratio; AFP, alpha fetoprotein; HBV, hepatitis B virus; HCV, hepatitis C virus; CTC, circulating tumor cells; CI, confidence interval; HR, hazard ratio; UV, univariable; MV, multivariable. *Those variables found significant at p < 0.05 in univariable analyses were entered into multivariable Cox-regression analyses.

Supplementary Table 2 Univariate and multivariate Cox regression analysis was used to identify independent risk factors for OS in overall patients. AST, aspartate aminotransferase; ALT, alanine aminotransferase; GGT, gamma glutamyl transpeptidase; ALP, alkaline phosphatase; Alb, albumin; TIBL, total bilirubin; DIBL, direct bilirubin; CR, creatinine; INR, international normalized ratio; AFP, alpha fetoprotein; HBV, hepatitis B virus; HCV, hepatitis C virus; CTC, circulating tumor cells; CI, confidence interval; HR, hazard ratio; UV, univariable; MV, multivariable. *Those variables found significant at p < 0.05 in univariable analyses were entered into multivariable Cox-regression analyses.

Supplementary Table 3 Univariate and multivariate Cox regression analysis was used to identify independent risk factors for RFS in patients with HCC ≤ 3 cm. AST, aspartate aminotransferase; ALT, alanine aminotransferase; GGT, gamma glutamyl transpeptidase; ALP, alkaline phosphatase; Alb, albumin; TIBL, total bilirubin; DIBL, direct bilirubin; CR, creatinine; INR, international normalized ratio; AFP, alpha fetoprotein; HBV, hepatitis B virus; HCV, hepatitis C virus; CTC, circulating tumor cells; CI, confidence interval; HR, hazard ratio; UV, univariable; MV, multivariable. *Those variables found significant at p < 0.05 in univariable analyses were entered into multivariable Cox-regression analyses.

Supplementary Table 4 Univariate and multivariate Cox regression analysis was used to identify independent risk factors for OS in patients with HCC ≤ 3 cm. AST, aspartate aminotransferase; ALT, alanine aminotransferase; GGT, gamma glutamyl transpeptidase; ALP, alkaline phosphatase; Alb, albumin; TIBL, total bilirubin; DIBL, direct bilirubin; CR, creatinine; INR, international normalized ratio; AFP, alpha fetoprotein; HBV, hepatitis B virus; HCV, hepatitis C virus; CTC, circulating tumor cells; CI, confidence interval; HR, hazard ratio; UV, univariable; MV, multivariable. *Those variables found significant at p < 0.05 in univariable analyses were entered into multivariable Cox-regression analyses.

Supplementary Table 5 Univariate and multivariate Cox regression analysis was used to identify independent risk factors for RFS in patients with HCC ≥ 3 cm. AST, aspartate aminotransferase; ALT, alanine aminotransferase; GGT, gamma glutamyl transpeptidase; ALP, alkaline phosphatase; Alb, albumin; TIBL, total bilirubin; DIBL, direct bilirubin; CR, creatinine; INR, international normalized ratio; AFP, alpha fetoprotein; HBV, hepatitis B virus; HCV, hepatitis C virus; CTC, circulating tumor cells; CI, confidence interval; HR, hazard ratio; UV, univariable; MV, multivariable. *Those variables found significant at p < 0.05 in univariable analyses were entered into multivariable Cox-regression analyses.

Supplementary Table 6 Univariate and multivariate Cox regression analysis was used to identify independent risk factors for OS in patients with HCC > 3 cm. AST, aspartate aminotransferase; ALT, alanine aminotransferase; GGT, gamma glutamyl transpeptidase; ALP, alkaline phosphatase; Alb, albumin; TIBL, total bilirubin; DIBL, direct bilirubin; CR, creatinine; INR, international normalized ratio; AFP, alpha fetoprotein; HBV, hepatitis B virus; HCV, hepatitis C virus; CTC, circulating tumor cells; CI, confidence interval; HR, hazard ratio; UV, univariable; MV, multivariable. *Those variables found significant at p < 0.05 in univariable analyses were entered into multivariable Cox-regression analyses.
