# Supplementary material for: Combined analysis of preoperative circulating tumor cells status and tumor size to guide width of resection margin for solitary hepatocellular carcinoma
Source: Front Oncol. 2026 Oct 16;16:1810914. doi: 10.3389/fonc.2026.1810914 (PMC13720440; doi:10.3389/fonc.2026.1810914)
Supplement: Supplementary file 1 [file Supplementaryfile1.docx]

**Supplementary Material**

**Supplementary Fig 1** Survival outcomes of the entire cohort and tumor size-based subgroups divided by CTC status. Recurrence-free survival and overall survival curves for the entire cohort (**A, B**), patients with tumor size ≤ 3 cm **(C, D)**, and those with tumor size > 3 cm (**E, F**) divided by CTC status.

**Supplementary Fig 2** Analysis of the effect of CTC status on early and late postoperative recurrence entire cohort (**A**), patients with tumor size ≤ 3 cm (**B**), and those with tumor size > 3 cm (**C**) by landmark method.

**Supplementary Table 1** Univariate and multivariate Cox regression analysis was used to identify independent risk factors for RFS in overall patients

| Variables | HR comparison | | UV HR (95% *CI*) | UV p | MV HR (95% *CI*) | MV p* |
| --- | --- | --- | --- | --- | --- | --- |
| Age | ≥60 vs＜60 years | 1.066(0.788-1.442) | | 0.678 |  |  |
| Gender | Male vs. female | 0.808(0.588-1.112) | | 0.191 |  |  |
| HBV | Yes vs. no | 0.845(0.549-1.3) | | 0.444 |  |  |
| HCV | Yes vs. no | 1.534(0.833-2.825) | | 0.169 |  |  |
| Cirrhosis | Yes vs. no | 0.996(0.729-1.359) | | 0.978 |  |  |
| Child Pugh | B vs A | 1.021(0.647-1.61) | | 0.930 |  |  |
| ALT | ≥50 vs＜50 U/L | 0.717(0.494-1.041) | | 0.080 |  |  |
| AST | ≥40 vs＜40 U/L | 0.779(0.556-1.091) | | 0.146 |  |  |
| GGT | ≥45 vs＜45 U/L | 0.855(0.614-1.192) | | 0.355 |  |  |
| ALP | ≥40 vs＜40 U/L | 0.894(0.59-1.354) | | 0.596 |  |  |
| Alb | ≥35 vs＜35 g/L | 1.231(0.84-1.804) | | 0.287 |  |  |
| TBIL | ≥20.4 vs＜20.4umol/L | 0.975(0.674-1.41) | | 0.893 |  |  |
| CR | ≥80.4 vs＜80.4umol /L | 1.301(0.482-3.513) | | 0.603 |  |  |
| INR | ≥1.15 vs＜1.15 | 1.589(0.92-2.743) | | 0.097 |  |  |
| AFP | ≥400 vs＜400ng/mL | 1.972(1.463-2.658) | | 0.000 | 1.574(1.157-2.14) | 0.004 |
| Grade | I+II vs III+IV | 1.424(1.024-1.98) | | 0.036 | 1.406(1.007-1.963) | 0.046 |
| MVI | Yes vs No | 2.172(1.618-2.914) | | 0.000 | 1.56(1.139-2.137) | 0.006 |
| Size | ＞3 cm vs ≤ 3 cm | 1.778(1.304-2.425) | | 0.000 | 1.399(1.01-1.937) | 0.044 |
| Group | Narrow vs Wide | 0.591(0.428-0.815) | | 0.001 | 0.624(0.449-0.867) | 0.005 |
| CTC | (+) vs (-) | 2.172(1.624-2.905) | | 0.000 | 1.833(1.353-2.484) | 0.000 |

Abbreviations: AST aspartate aminotransferase, ALT alanine aminotransferase, GGT gamma glutamyl transpeptidase, ALP alkaline phosphatase, Alb albumin, TIBL total bilirubin, DIBL direct bilirubin, CR creatinine, INR international normalized ratio, AFP alpha fetoprotein, HBV hepatitis B virus, HCV hepatitis C virus, .CTC circulating tumor cells, CI confidence interval, HR hazard ratio, UV univariable, MV multivariable

*Those variables found significant at p < 0.05 in univariable analyses were entered into multivariable Cox-regression analyses

**Supplementary Table 2** Univariate and multivariate Cox regression analysis was used to identify independent risk factors for OS in overall patients

| Variables | HR comparison | | UV HR (95% *CI*) | UV p | MV HR (95% *CI*) | MV p* |
| --- | --- | --- | --- | --- | --- | --- |
| Age | ≥60 vs＜60 years | 0.924(0.608-1.405) | | 0.712 |  |  |
| Gender | Male vs. female | 0.957(0.604-1.517) | | 0.853 |  |  |
| HBV | Yes vs. no | 0.796(0.443-1.434) | | 0.448 |  |  |
| HCV | Yes vs. no | 1.542(0.674-3.528) | | 0.306 |  |  |
| Cirrhosis | Yes vs. no | 1.11(0.711-1.731) | | 0.647 |  |  |
| Child Pugh | B vs A | 1.62(0.944-2.781) | | 0.080 |  |  |
| ALT | ≥50 vs＜50 U/L | 1.336(0.852-2.093) | | 0.207 |  |  |
| AST | ≥40 vs＜40 U/L | 1.13(0.728-1.756) | | 0.585 |  |  |
| GGT | ≥45 vs＜45 U/L | 1.211(0.783-1.873) | | 0.390 |  |  |
| ALP | ≥40 vs＜40 U/L | 0.712(0.379-1.337) | | 0.291 |  |  |
| Alb | ≥35 vs＜35 g/L | 1.721(0.956-3.1) | | 0.070 |  |  |
| TBIL | ≥20.4 vs＜20.4umol/L | 1.137(0.686-1.885) | | 0.618 |  |  |
| CR | ≥80.4 vs＜80.4umol /L | 1.324(0.325-5.387) | | 0.696 |  |  |
| INR | ≥1.15 vs＜1.15 | 1.278(0.558-2.927) | | 0.561 |  |  |
| AFP | ≥400 vs＜400ng/mL | 2.852(1.887-4.312) | | 0.000 | 1.972(1.282-3.032) | 0.002 |
| Grade | I+II vs III+IV | 2.32(1.522-3.536) | | 0.000 | 1.945(1.266-2.989) | 0.002 |
| MVI | Yes vs No | 3.286(2.177-4.96) | | 0.000 | 1.852(1.201-2.855) | 0.005 |
| Size | ＞3 cm vs ≤3 cm | 4.204(2.418-7.311) | | 0.000 | 2.918(1.652-5.154) | 0.000 |
| Group | Narrow vs Wide | 0.424(0.258-0.696) | | 0.001 | 0.501(0.301-0.834) | 0.008 |
| CTC | (+) vs (-) | 3.127(2.054-4.761) | | 0.000 | 2.247(1.443-3.496) | 0.000 |

Abbreviations: AST aspartate aminotransferase, ALT alanine aminotransferase, GGT gamma glutamyl transpeptidase, ALP alkaline phosphatase, Alb albumin, TIBL total bilirubin, DIBL direct bilirubin, CR creatinine, INR international normalized ratio, AFP alpha fetoprotein, HBV hepatitis B virus, HCV hepatitis C virus, .CTC circulating tumor cells, CI confidence interval, HR hazard ratio, UV univariable, MV multivariable

*Those variables found significant at p < 0.05 in univariable analyses were entered into multivariable Cox-regression analyses

**Supplementary Table 3** Univariate and multivariate Cox regression analysis was used to identify independent risk factors for RFS in patients with HCC ≤ 3 cm.

| Variables | HR comparison | UV HR (95% *CI*) | | UV p | MV HR (95% *CI*) | MV p* |
| --- | --- | --- | --- | --- | --- | --- |
| Age | ≥60 vs＜60 years | | 1.441(0.883-2.352) | 0.144 |  |  |
| Gender | Male vs. female | | 0.802(0.478-1.344) | 0.402 |  |  |
| HBV | Yes vs. no | | 1.145(0.569-2.304) | 0.705 |  |  |
| HCV | Yes vs. no | | 2.013(0.807-5.025) | 0.134 |  |  |
| Cirrhosis | Yes vs. no | | 0.872(0.538-1.415) | 0.580 |  |  |
| Child Pugh | B vs A | | 0.754(0.346-1.644) | 0.477 |  |  |
| ALT | ≥50 vs＜50 U/L | | 0.581(0.324-1.042) | 0.068 |  |  |
| AST | ≥40 vs＜40 U/L | | 0.798(0.476-1.337) | 0.391 |  |  |
| GGT | ≥45 vs＜45 U/L | | 0.875(0.514-1.489) | 0.623 |  |  |
| ALP | ≥40 vs＜40 U/L | | 0.519(0.258-1.042) | 0.065 |  |  |
| Alb | ≥35 vs＜35 g/L | | 0.953(0.532-1.709) | 0.873 |  |  |
| TBIL | ≥20.4 vs＜20.4umol/L | | 1.567(0.92-2.671) | 0.099 |  |  |
| CR | ≥80.4 vs＜80.4umol /L | | 0.824(0.114-5.947) | 0.848 |  |  |
| INR | ≥1.15 vs＜1.15 | | 2.049(0.973-4.315) | 0.059 |  |  |
| AFP | ≥400 vs＜400ng/mL | | 1.648(1.014-2.68) | 0.044 | 1.375(0.828-2.285) | 0.021 |
| Grade | I+II vs III+IV | | 1.046(0.537-2.039) | 0.894 |  |  |
| MVI | Yes vs No | | 2.068(1.194-3.581) | 0.010 | 1.756(0.994-3.103) | 0.0524 |
| Group | Narrow vs Wide | | 0.867(0.538-1.398) | 0.559 |  |  |
| CTC | (+) vs (-) | | 1.755(1.096-2.812) | 0.019 | 1.517(0.929-2.477) | 0.0957 |

Abbreviations: AST aspartate aminotransferase, ALT alanine aminotransferase, GGT gamma glutamyl transpeptidase, ALP alkaline phosphatase, Alb albumin, TIBL total bilirubin, DIBL direct bilirubin, CR creatinine, INR international normalized ratio, AFP alpha fetoprotein, HBV hepatitis B virus, HCV hepatitis C virus, .CTC circulating tumor cells, CI confidence interval, HR hazard ratio, UV univariable, MV multivariable

*Those variables found significant at p < 0.05 in univariable analyses were entered into multivariable Cox-regression analyses

**Supplementary Table 4** Univariate and multivariate Cox regression analysis was used to identify independent risk factors for OS in patients with HCC ≤ 3 cm.

| Variables | HR comparison | | UV HR (95% *CI*) | UV p | MV HR (95% *CI*) | MV p* |
| --- | --- | --- | --- | --- | --- | --- |
| Age | ≥60 vs＜60 years | 1.176(0.557-2.481) | | 0.670 |  |  |
| Gender | Male vs. female | 1.972(0.686-5.665) | | 0.207 |  |  |
| HBV | Yes vs. no | 1.07(0.372-3.073) | | 0.900 |  |  |
| HCV | Yes vs. no | 1.869(0.44-7.931) | | 0.397 |  |  |
| Cirrhosis | Yes vs. no | 1.958(0.8-4.791) | | 0.141 |  |  |
| Child Pugh | B vs A | 1.431(0.547-3.746) | | 0.465 |  |  |
| ALT | ≥50 vs＜50 U/L | 1.207(0.563-2.587) | | 0.628 |  |  |
| AST | ≥40 vs＜40 U/L | 1.456(0.7-3.029) | | 0.315 |  |  |
| GGT | ≥45 vs＜45 U/L | 1.194(0.546-2.608) | | 0.657 |  |  |
| ALP | ≥40 vs＜40 U/L | 0.43(0.13-1.418) | | 0.166 |  |  |
| Alb | ≥35 vs＜35 g/L | 1.378(0.526-3.606) | | 0.514 |  |  |
| TBIL | ≥20.4 vs＜20.4umol/L | 1.638(0.7-3.833) | | 0.255 |  |  |
| CR | ≥80.4 vs＜80.4umol /L | 0(0-Inf) | | 0.997 |  |  |
| INR | ≥1.15 vs＜1.15 | 1.726(0.52-5.732) | | 0.373 |  |  |
| AFP | ≥400 vs＜400ng/mL | 2.78(1.352-5.718) | | 0.005 | 2.454(1.16-5.192) | 0.019 |
| Grade | I+II vs III+IV | 1.068(0.372-3.065) | | 0.903 |  |  |
| MVI | Yes vs No | 2.407(1.06-5.467) | | 0.036 | 2.337(1.186-4.296) | 0.004 |
| Group | Narrow vs Wide | 0.757(0.354-1.62) | | 0.474 |  |  |
| CTC | (+) vs (-) | 1.855(0.892-3.856) | | 0.098 |  |  |

Abbreviations: AST aspartate aminotransferase, ALT alanine aminotransferase, GGT gamma glutamyl transpeptidase, ALP alkaline phosphatase, Alb albumin, TIBL total bilirubin, DIBL direct bilirubin, CR creatinine, INR international normalized ratio, AFP alpha fetoprotein, HBV hepatitis B virus, HCV hepatitis C virus, .CTC circulating tumor cells, CI confidence interval, HR hazard ratio, UV univariable, MV multivariable

*Those variables found significant at p < 0.05 in univariable analyses were entered into multivariable Cox-regression analyses

**Supplementary Table 5** Univariate and multivariate Cox regression analysis was used to identify independent risk factors for RFS in patients with HCC ≥ 3 cm.

| Variables | HR comparison | UV HR (95% *CI*) | | UV p | MV HR (95% *CI*) | MV p* |
| --- | --- | --- | --- | --- | --- | --- |
| Age | ≥60 vs＜60 years | | 0.862(0.586-1.269) | 0.452 |  |  |
| Gender | Male vs. female | | 0.829(0.552-1.244) | 0.365 |  |  |
| HBV | Yes vs. no | | 0.644(0.372-1.115) | 0.116 |  |  |
| HCV | Yes vs. no | | 1.243(0.543-2.843) | 0.606 |  |  |
| Cirrhosis | Yes vs. no | | 1.083(0.72-1.631) | 0.701 |  |  |
| Child Pugh | B vs A | | 1.231(0.701-2.163) | 0.470 |  |  |
| ALT | ≥50 vs＜50 U/L | | 0.662(0.363-1.208) | 0.179 |  |  |
| AST | ≥40 vs＜40 U/L | | 0.769(0.492-1.203) | 0.250 |  |  |
| GGT | ≥45 vs＜45 U/L | | 0.821(0.537-1.258) | 0.365 |  |  |
| ALP | ≥40 vs＜40 U/L | | 1.537(0.915-2.581) | 0.104 |  |  |
| Alb | ≥35 vs＜35 g/L | | 1.463(0.88-2.431) | 0.142 |  |  |
| TBIL | ≥20.4 vs＜20.4umol/L | | 0.67(0.399-1.125) | 0.130 |  |  |
| CR | ≥80.4 vs＜80.4umol /L | | 1.599(0.507-5.048) | 0.423 |  |  |
| INR | ≥1.15 vs＜1.15 | | 1.903(0.959-3.774) | 0.066 |  |  |
| AFP | ≥400 vs＜400ng/mL | | 2.091(1.425-3.067) | 0.000 | 1.675(1.13-2.484) | 0.010 |
| Grade | I+II vs III+IV | | 1.205(1.108-1.863) | 0.040 | 1.123(1.080-1.863) | 0.043 |
| MVI | Yes vs No | | 2.063(1.412-3.015) | 0.000 | 1.568(1.058-2.324) | 0.025 |
| Group | Narrow vs Wide | | 0.438(0.282-0.682) | 0.000 | 0.5(0.318-0.787) | 0.003 |
| CTCs | (+) vs (-) | | 2.324(1.581-3.414) | 0.000 | 2.036(1.363-3.041) | 0.001 |

Abbreviations: AST aspartate aminotransferase, ALT alanine aminotransferase, GGT gamma glutamyl transpeptidase, ALP alkaline phosphatase, Alb albumin, TIBL total bilirubin, DIBL direct bilirubin, CR creatinine, INR international normalized ratio, AFP alpha fetoprotein, HBV hepatitis B virus, HCV hepatitis C virus, .CTC circulating tumor cells, CI confidence interval, HR hazard ratio, UV univariable, MV multivariable

*Those variables found significant at p < 0.05 in univariable analyses were entered into multivariable Cox-regression analyses

**Supplementary Table 6** Univariate and multivariate Cox regression analysis was used to identify independent risk factors for OS in patients with HCC > 3 cm.

| Variables | HR comparison | UV HR (95% *CI*) | | UV p | MV HR (95% *CI*) | MV p* |
| --- | --- | --- | --- | --- | --- | --- |
| Age | ≥60 vs＜60 years | | 0.818(0.492-1.36) | 0.439 |  |  |
| Gender | Male vs. female | | 0.78(0.46-1.321) | 0.355 |  |  |
| HBV | Yes vs. no | | 0.637(0.314-1.293) | 0.212 |  |  |
| HCV | Yes vs. no | | 1.341(0.486-3.702) | 0.571 |  |  |
| Cirrhosis | Yes vs. no | | 0.866(0.514-1.459) | 0.589 |  |  |
| Child Pugh | B vs A | | 1.689(0.878-3.249) | 0.117 |  |  |
| ALT | ≥50 vs＜50 U/L | | 0.965(0.476-1.958) | 0.921 |  |  |
| AST | ≥40 vs＜40 U/L | | 0.996(0.57-1.741) | 0.988 |  |  |
| GGT | ≥45 vs＜45 U/L | | 1.182(0.698-2.002) | 0.535 |  |  |
| ALP | ≥40 vs＜40 U/L | | 1.088(0.517-2.288) | 0.825 |  |  |
| Alb | ≥35 vs＜35 g/L | | 1.945(0.924-4.094) | 0.080 |  |  |
| TBIL | ≥20.4 vs＜20.4umol/L | | 0.93(0.495-1.746) | 0.820 |  |  |
| CR | ≥80.4 vs＜80.4umol /L | | 1.905(0.465-7.806) | 0.370 |  |  |
| INR | ≥1.15 vs＜1.15 | | 1.875(0.806-4.36) | 0.144 |  |  |
| AFP | ≥400 vs＜400ng/mL | | 2.658(1.602-4.411) | 0.000 | 1.891(1.122-3.189) | 0.017 |
| Grade | I+II vs III+IV | | 1.54(1.003-2.624) | 0.013 | 1.44(1.302-2.125) | 0.043 |
| MVI | Yes vs No | | 3.257(1.911-5.553) | 0.000 | 2.239(1.292-3.88) | 0.004 |
| Group | Wide vs Narrow | | 0.291(0.148-0.573) | 0.000 | 0.382(0.19-0.766) | 0.007 |
| CTCs | (+) vs (-) | | 3.712(2.135-6.455) | 0.000 | 2.862(1.603-5.108) | 0.000 |

Abbreviations: AST aspartate aminotransferase, ALT alanine aminotransferase, GGT gamma glutamyl transpeptidase, ALP alkaline phosphatase, Alb albumin, TIBL total bilirubin, DIBL direct bilirubin, CR creatinine, INR international normalized ratio, AFP alpha fetoprotein, HBV hepatitis B virus, HCV hepatitis C virus, .CTC circulating tumor cells, CI confidence interval, HR hazard ratio, UV univariable, MV multivariable

*Those variables found significant at p < 0.05 in univariable analyses were entered into multivariable Cox-regression analyses
